# Resource partitioning between ungulate populations in arid environments

**DOI:** 10.1002/ece3.2218

**Published:** 2016-08-17

**Authors:** Robert S. C. Cooke, Tim Woodfine, Marie Petretto, Thomas H. G. Ezard

**Affiliations:** ^1^ Centre for Biological SciencesUniversity of SouthamptonLife Sciences Building (B85)Highfield CampusSouthamptonSO17 1BJUK; ^2^ Marwell WildlifeThompson's LaneColden CommonWinchesterHampshireSO21 1JHUK; ^3^ School of Ocean and Earth SciencesNational Oceanography Centre SouthamptonUniversity of SouthamptonSouthamptonSO17 1BJUK

**Keywords:** Desert, distance sampling, dorcas gazelle, North Africa, reintroduction, scimitar‐horned oryx

## Abstract

Herbivores are major drivers of ecosystem structure, diversity, and function. Resilient ecosystems therefore require viable herbivore populations in a sustainable balance with environmental resource availability. This balance is becoming harder to achieve, with increasingly threatened species reliant on small protected areas in increasingly harsh and unpredictable environments. Arid environments in North Africa exemplify this situation, featuring a biologically distinct species assemblage exposed to extreme and volatile conditions, including habitat loss and climate change‐associated threats. Here, we implement an integrated likelihood approach to relate scimitar‐horned oryx (*Oryx dammah*) and dorcas gazelle (*Gazella dorcas*) density, via dung distance sampling, to habitat, predator, and geographic correlates in Dghoumes National Park, Tunisia. We show how two threatened sympatric ungulates partition resources on the habitat axis, exhibiting nonuniform responses to the same vegetation gradient. Scimitar‐horned oryx were positively associated with plant species richness, selecting for vegetated ephemeral watercourses (wadis) dominated by herbaceous cover. Conversely, dorcas gazelle were negatively associated with vegetation density (herbaceous height, litter cover, and herbaceous cover), selecting instead for rocky plains with sparse vegetation. We suggest that adequate plant species richness should be a prerequisite for areas proposed for future ungulate reintroductions in arid and semi‐arid environments. This evidence will inform adaptive management of reintroduced ungulates in protected environments, helping managers and planners design sustainable ecosystems and effective conservation programs.

## Introduction

Effective conservation management is essential for the dynamic arid regions of the world (Durant et al. 2014). Arid environments cover 17% of the world's land mass and harbor 25% of terrestrial vertebrate species (Mace et al. 2005; Safriel et al. 2005), including charismatic and threatened species such as antelopes (Durant et al. 2014). Global data from the IUCN Antelope Specialist Group show that 27% of antelope species are threatened with extinction; however, this rises to 89% when only arid‐adapted antelope are considered (Mésochina and Cooke 2015). Desertic environments are characterized by low biomass and vegetative cover relative to more mesic systems, with high spatiotemporal variability driven by pulses of resource saturation (Illius and O'Connor 2000; Schwinning and Sala 2004). These pulses drive stochastic events, including unpredicted population declines (Illius and O'Connor 2000). Small protected areas, where many threatened populations live, intensify these threats (Islam et al. 2010; Durant et al. 2015). Arid ecosystems can therefore provide important insights into extinction risk for dynamic, yet constrained, environments.

Herbivores are major drivers of ecosystem structure, diversity, and function (Danell et al. 2006). Viable herbivore populations depend upon their distribution and abundance and the fitness of individuals, which are often determined by habitat and resource selection (Gaillard et al. 2010; DeCesare et al. 2014; Boyce et al. 2016). These resource selection decisions shape the capacity of herbivores to respond to their environment and depend upon innate‐specific ecological preferences determined by multiple synergistic factors including predation, climatic conditions, vegetation, terrain features, and competition, all mediated by interspecific differences in body size and life history (Kittle et al. 2008). Understanding which factors drive these resource decisions is vital for effective species‐ and landscape‐level management (Cromsigt and Olff 2006; Kittle et al. 2008). This is particularly acute for arid ecosystems, where populations must cope with low resource availability, resource pulses, and stochastic events, leading to disparate herbivore responses including resource partitioning (Illius and O'Connor 2000; Ostfeld and Keesing 2000). Techniques that robustly quantify ungulate density and its determining factors are therefore a foundational management tool (Marques et al. 2001; Laing et al. 2003).

The arid North African Sahelo‐Saharan region contains a distinct species assemblage (Burgess et al. 2006) subject to high levels of present and future threats (Thomas 2008), yet has attracted very little scientific attention (Durant et al. 2014) compared to African savannahs (Darmon et al. 2012). The magnitude and velocity of climate change in North Africa are predicted to be strong and fast, including more frequent droughts and changes in rainfall patterns (Thomas 2008; Loarie et al. 2009). These changes will impact vegetation and habitat structure and, in turn, influence resource decisions and competitive interactions between species (Post and Pedersen 2008). Habitats have substantial influence on herbivore distribution and abundance (Boyce et al. 2016), and species' resource decisions may vary considerably depending on the focal species' behavior and its perception of habitat (Tews et al. 2004). Despite this crucial role, habitat type is rarely quantified directly and is instead often categorized subjectively based on inference from structural parameters or dominant species (e.g., Cromsigt et al. 2009; Darmon et al. 2012). Here, we complement a subjective assessment with a quantitative habitat classification, where the focal species' density and resource preferences convey their perception of habitat.

We develop the themes identified in studies of Asiatic wild ass (*Equus hemionus*) and dorcas gazelle (*Gazella dorcas*) in Israel (Henley et al. 2007); six savannah ungulates in South Africa (Cromsigt et al. 2009); and dorcas gazelle in Senegal (Abáigar et al. 2013). We employ a novel integrated likelihood approach for indirect (dung) distance sampling (Oedekoven et al. 2013), to relate, for the first time, environmental correlates to ungulate density in an arid environment. By linking species decisions from the feeding patch level, through habitat types to the dynamic arid landscape, this quantification of scimitar‐horned oryx (*Oryx dammah*) and dorcas gazelle density and its determining factors aims to improve our understanding of resource partitioning and habitat selection in infrequently studied environments.

## Methods

### Study site

**Figure 1:**
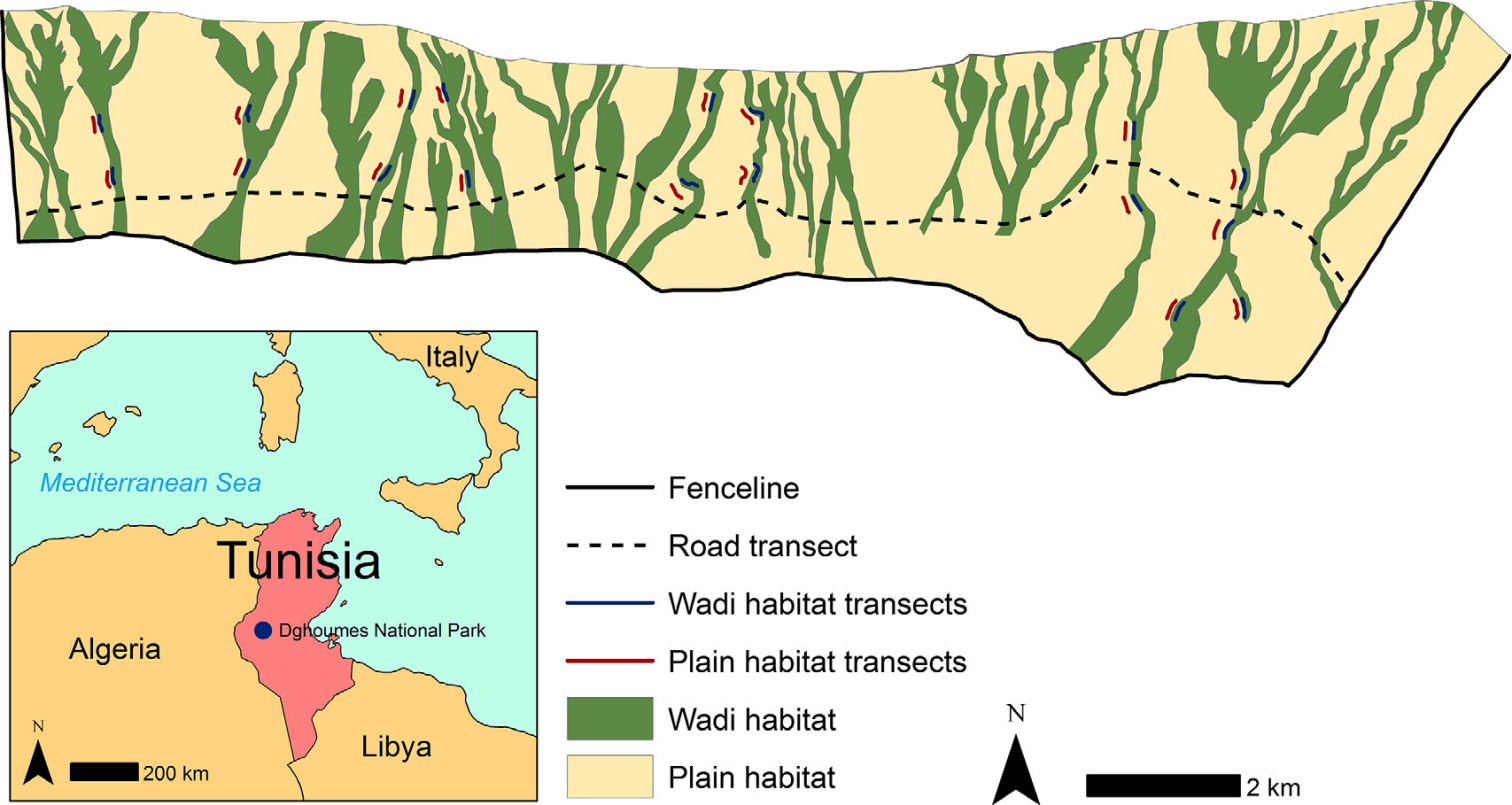
Habitat map of Dghoumes National Park and its location within Tunisia (inset). Dghoumes is unfenced to the north, with the mountains acting as a physical barrier. The locations of the direct (road) and indirect (wadi and plain) transects are also presented. Two pairs of transects were placed in the southeast of the reserve to account for a distinct physiognomic dune area.

The fenced section of Dghoumes National Park, Tunisia (34°03′N, 8°33′E), comprises two distinct areas of topography: an intermediate plain consisting of continental subdesert steppe, marked by a series of wadis, and a mountain region to the north (Le Houérou 2001; Woodfine et al. 2009). Although both species had access to and made occasional use of the adjoining mountains (R. S. C. Cooke, pers. obs.), we restricted our investigation to the plain (3800 ha; Fig. 1). Fieldwork was conducted during March and April 2014. We surveyed two reintroduced ungulate populations: the large‐bodied (≈150 kg) scimitar‐horned oryx and the small‐bodied (≈15 kg) dorcas gazelle (Kingdon et al. 2013), hereafter oryx and gazelle. Gazelle were reintroduced to Dghoumes in 2002 and by 2012 numbered approximately 60 individuals; oryx were reintroduced in 2008 and reached approximately 75 individuals by 2012 (M. Petretto, pers. obs; Woodfine et al. 2009).

### Indirect distance sampling

We surveyed 18 pairs of 200 × 8 m strip transects (Fig. 1). Pairs consisted of one transect in wadi habitat and a parallel transect 100 m to the west in plain habitat. The 100‐m spacing was approximately half the distance between the sampled wadi with the nearest neighboring wadi, that is, equidistant between the closest two wadis, to ensure the plain transects fell in the adjoining interwadi plain for all locations. Eight wadi systems (the major wadis across Dghoumes and their adjoining plains) were chosen to represent the regions substrate and vegetation gradients. Perpendicular distances were recorded from the line to the center of all fecal pellet events within the sampling band. Pellet events were defined as a group of at least 10 pellets of consistent age and size. Although ungulates do not always defecate where they graze, that is, in the actual feeding patch, they generally defecate in the same locality as where they forage (Cromsigt et al. 2009). The wide sampling strip aimed to account for this and to reduce the potential for bias from edge effects (Marques et al. 2001).

A total of 825 pellet events were recorded for oryx (640) and gazelle (185). Of these, 65 pellet events (12 oryx and 53 gazelle) were classified as territorial clusters of feces known as middens (Attum and Mahmoud 2012). Middens do not reflect resource use with their location driven by territorial factors (Attum and Mahmoud 2012), and so were excluded from analysis, leaving 628 oryx and 132 gazelle pellet events from which density was calculated in DISTANCE 6.2 (Thomas et al. 2010).

### Population estimates

As a comparative density estimate, we carried out direct distance sampling along the 14 km central road six times during the survey period (Fig. 1). For each observation, we recorded species, number of individuals, radial angle and sight distance at first contact. This was also compared to previous sweep census estimates; in which a line of observers (park guards and MP; spaced so that each only views in one direction and can see the next observer) crossed the park from the western to the eastern boundary (sensu Bowland and Perrin 1994). Population estimates were generated from direct and indirect data in DISTANCE 6.2 at the site and habitat level. Indirect data require estimates of defecation rate (Appendix S2) and decay rate (Appendix S3). The decay rates demonstrate that a long‐term dataset (490–520 days) on habitat use is produced from a short survey period in arid environments.

### Vegetation

Vegetation sampling was designed to quantify relevant environmental variation in vegetative structure, forage availability, and water availability (Voeten and Prins 1999; Henley et al. 2007). Eleven vegetation quadrats (1 m^2^) were placed 20 m apart along the center line of the transects. Structure was determined by visually estimating percentage cover of habitat components: rock, litter (dead vegetative matter), herbaceous, shrub and tree (Tabeni and Ojeda 2005). Plant species richness [species were identified by RC, MP, and park guards following Ozenda (2004)] and the mean height of each plant stratum were also recorded (Voeten and Prins 1999; Tabeni and Ojeda 2005). A smaller biomass quadrat (0.063 m^2^) was placed randomly inside each vegetation quadrat. This quadrat was clipped to ground level and sorted into woody and nonwoody biomass. The nonwoody portion was weighed initially, then dried, and weighed repeatedly until the mass reached an asymptote to calculate percentage water content.

### Predation

The combined relative abundance of the predators, African golden wolf (*Canis anthus*), Rüppell's fox (*Vulpes rueppellii*), and red fox (*Vulpes vulpes*) (Yom‐Tov et al. 1995; Gilbert and Woodfine 2004), was approximated by the number of Canidae fecal scats within each transect (total of 38 scats recorded).

### Integrated likelihood approach

A new integrated likelihood approach (Oedekoven et al. 2013) was applied to the environmental data collected. For this, we modeled 17 predictor variables and hypothesized their effect (Table 1). Vegetative strata were defined by their physical and functional characteristics: herbaceous (vegetation consisting entirely of nonwoody biomass), shrub (vegetation with an average height of <1 m, consisting of both woody and nonwoody biomass), and tree (vegetation consisting of both woody and nonwoody biomass, with an average height of >1 m). These were quantified in both the vertical dimensions, where different grazers specialize on different heights (Farnsworth et al. 2002) and the horizontal dimension, where ungulates demonstrate patch‐specific use of resources (Turner et al. 1997).

**Table 1:** Predictor variables and their anticipated effect on the response variables: scimitar‐horned oryx and dorcas gazelle density

Driver	Hypothesis	Reference(s)
Habitat type (wadi/plain)	We expect both species to preferentially utilize the wadi habitat, due to its greater vegetation and shade	Beudels et al. (2005)
Wadi location	We expect the larger oryx to use a higher proportion of the landscape and therefore be less dependent on specific wadi systems than the smaller gazelle	Cromsigt et al. (2009)
North–south gradient	A topographic gradient from the elevated northern transects to the more saline south. We expect both species to prefer the northern regions, which have greater access to the mountains. This response may be more intense for gazelle, as they avoid consuming halophytic plants	Yom‐Tov et al. (1995)
East–west gradient	A substrate gradient from sand in the east to gravel in the west. We would expect oryx to prefer the east, with its enlarged hooves and gazelle the west, due to its smaller hooves	Yom‐Tov et al. (1995), Beudels et al. (2005)
Rock cover	A fine scale representation of the substrate gradient (see east–west gradient)	Yom‐Tov et al. (1995), Beudels et al. (2005)
Litter cover	High litter cover correlates with high vegetation availability and density and therefore forage and shade. A positive association is expected	Beudels et al. (2005)
Nonwoody biomass	Equates to forage availability, we expect it to be positively related to ungulate density in this resource‐limited environment	
Woody biomass	Characterizes browse availability, as both species demonstrate flexible foraging strategies, we predict positive associations	Beudels et al. (2005)
Herbaceous cover and height	Equates to graze, which is important for both species and particularly for oryx, who are primarily grazers	Gilbert and Woodfine (2004)
Shrub cover and height	Provides low‐growing browse and shade, especially for gazelle which prefer shallow depressions protected by shrubs	Yom‐Tov et al. (1995)
Tree cover and height	Trees function as shade providers, which is a habitat characteristic, that is, known to be important for gazelle and oryx. This shade often leads to high concentrations of annual plants under tree canopies; therefore, we expect both species to select for areas of high tree cover/height	Yom‐Tov et al. (1995), Beudels et al. (2005), Attum and Mahmoud (2012)
Plant water content	Gazelle and oryx do not rely on free water, but are strongly dependent on moisture‐rich plants; thus, we expect positive relationships	Kingdon et al. (2013)
Plant species richness	Plant species richness represents the opportunity to select a diet of appropriate quality. We therefore expect both species to select for high plant species richness in order to maximize nutrient intake	Freeland and Janzen (1974), Westoby (1978), Henley et al. (2007)
Predation	We expect both species to avoid areas with high predator abundance, especially gazelle, as smaller herbivores experience higher predation pressure than larger herbivores	Sinclair et al. (2003), Kittle et al. (2008)

The integrated likelihood approach can accommodate surveys with nonrandom placement of transects and imperfect detectability. A generalized linear mixed‐effect model was used to simultaneously estimate density via a log link with a Poisson error structure (see Appendix S1) and a global half‐normal detection function in three distance intervals away from the center line (0–1.33; 1.33–2.67; 2.67–4 m). This detection function produced a lower Akaike information criterion corrected for small sample size (AICc) and a more stable model (more consistent Hessian matrix) compared to a hazard‐rate detection. The model was implemented in R version 3.1.1 (R Core Team 2014).

The large number of possible models prevented meaningful stepwise model selection procedures. Instead, we first tested all univariate models of predictor variables and then generated multivariate models of the best predictor variables (based on AICc for the univariate models; Burnham and Anderson 2002) with all possible two‐way interactions. We used Akaike weights (*w*_i_) to determine the relative probability of each candidate model being the “correct” model (Mazerolle 2006). This allowed a 95% confidence set of best‐ranked models to be established, whereby models were included until cumulative *w*_i_ reached 0.95 (Burnham and Anderson 2002). Model average coefficients were calculated across the entire candidate set for each of the major predictors (selected based on summed *w*_i_) incorporating model uncertainty (Mazerolle 2006) and are provided in the text.

### Habitat classification

A priori habitat structure was categorized according to physiognomic features, whereby vegetation characteristics were resolved on the ground and then applied across an extent as determined by satellite imagery. To produce a posteriori classified habitats, we used Ward hierarchical clustering, to relate oryx and gazelle density to key predictor variables from the integrated approach for all 36 transects. The goal of these techniques was to quantify habitat subjectively (a priori) and objectively (a posteriori, i.e., from the ungulate's perspective; Krasnov et al. 1996).

As a measure of habitat selection, we calculated standardized selection ratios (Manly et al. 2002). These can be interpreted as the probability that a species will select a habitat if all were equally available. These ratios were utilized to identify key resource areas (ratio ≥0.5; Illius and O'Connor 2000). Diversity of habitat use (H') was then calculated as a Shannon–Wiener diversity index, utilizing the selection ratios as proportions of habitat use (Cromsigt et al. 2009).

## Results

### Population estimates

We undertook three population census methods, each of which returned similar estimates of abundance (Table 2). The two direct methods provide snapshots of gazelle and oryx abundance during the survey; the indirect approach quantifies average density over a period corresponding to the mean time to decay (490–520 days; Laing et al. 2003).

**Table 2:** Indirect and direct population estimates

	Indirect (distance sampling)^a^^,^ b		Direct (distance sampling)^a^^,^ c		Direct (sweep census)^d^
	Density	CI	Density	CI	Density
Scimitar‐horned oryx	107	71–155	103	61–171	75
Dorcas gazelle	49	27–91	53	11–228	60

a Indirect and direct distance sampling estimates generated in DISTANCE with 95% confidence intervals.

b Estimated from a sample size of 628 oryx and 132 gazelle pellet events.

c Estimated from a sample size of 163 oryx and 11 gazelle sightings.

d Sweep census carried out in 2012 (MP).

### Integrated likelihood approach

Four of the 22 candidate models (Appendix S4) were included in the 95% confidence set for scimitar‐horned oryx density (Table 3). Models 1 and 2 had effectively equivalent support (ΔAICc < 2) and therefore interpreted as equally valid predictors of variation in oryx density. Rock cover, plant species richness, and habitat type were, in that order (based on summed *w*_i_), the major predictors of oryx density; including the interactions between these predictors did not improve the model. The model‐averaged coefficient was negative for rock cover (−0.031, 95% CI: −0.017, −0.046), positive for plant species richness (0.208, 95% CI: 0.135, 0.281), and negative for habitat type (−0.691, 95% CI: −0.726, −0.657). Oryx density was therefore higher in the wadi habitat, areas of low rock cover, and/or high plant species richness.

**Table 3:** The confidence set (cumulative *w*_i_ ≥ 0.95) and global null model for scimitar‐horned oryx (based on 628 pellet events), with the number of parameters (*k*), AICc, ΔAICc, and Akaike weights (*w*_i_). The density model included the covariates, in addition to the intercept *β*_*0*_ and the random effect *b*_*j*_ (wadi system)

ID	Density model	*k*	AICc	ΔAICc	*w*_i_
1	*β*_*0*_ * + b*_*j*_ + Rock cover + Plant species richness	5	524.718	0.000	0.488
2	*β*_*0*_ + *b*_j_ + Rock cover + Habitat type (wadi/plain)	5	525.626	0.901	0.310
3	*β*_*0*_ + *b*_j_ + Rock cover + Herbaceous height	5	528.079	3.362	0.091
4	*β*_*0*_ + *b*_j_ + Rock cover + Herbaceous cover	5	528.752	4.034	0.065
5	*β*_*0*_ + *b*_j_ (Global null model)	3	555.984	31.267	0.000

For dorcas gazelle, 16 of the 19 candidate models (Appendix S4) were included in the 95% confidence set (Table 4), indicating lower discriminatory power than for oryx. Herbaceous height, litter cover, and herbaceous cover were the most influential variables, but less dominant than the analogous oryx models. Models 1–4 explained the same qualitative amounts of variation in gazelle density (ΔAICc < 2). Herbaceous height (−0.053, 95% CI: −0.095, −0.012), litter cover (−0.073, 95% CI: −0.083, −0.062), and herbaceous cover (−0.036, 95% CI: −0.040, −0.033) all had negative effects on gazelle density, which was therefore highest in areas of low herbaceous height and cover, with low litter cover.

**Table 4:** The confidence set (cumulative *w*_i_ ≥ 0.95) and global null model for dorcas gazelle (based on 132 pellet events), with the number of parameters (*k*), AICc, ΔAICc, and Akaike weights (*w*_i_). The density model included the covariates, in addition to the intercept *β*_*0*_ and the random effect *b*_*j*_ (wadi system)

ID	Density model	*k*	AICc	ΔAICc	*w*_i_
1	*β*_*0*_ + *b*_j_ + Herbaceous height × Litter cover^a^	6	318.246	0.000	0.184
2	*β*_*0*_ + *b*_j_ + Herbaceous height	4	318.781	0.535	0.141
3	*β*_*0*_ + *b*_j_ + Litter cover	4	319.157	0.911	0.117
4	*β*_*0*_ + *b*_j_ + Herbaceous cover	4	319.265	1.019	0.111
5	*β*_*0*_ + *b*_j_ + Nonwoody biomass	4	320.477	2.231	0.060
6	*β*_*0*_ + *b*_j_ (Global null model)	3	320.800	2.554	0.051
7	*β*_*0*_ + *b*_j_ + Plant water content	4	320.933	2.687	0.048
8	*β*_*0*_ + *b*_j_ + East–west gradient	4	321.591	3.345	0.035
9	*β*_*0*_ + *b*_j_ + Tree cover	4	321.782	3.536	0.031
10	*β*_*0*_ + *b*_j_ + Habitat type (wadi/plain)	4	321.890	3.644	0.030
11	*β*_*0*_ + *b*_j_ + North–south gradient	4	321.905	3.659	0.030
12	*β*_*0*_ + *b*_j_ + Tree height	4	322.089	3.843	0.027
13	*β*_*0*_ + *b*_j_ + Shrub cover	4	322.140	3.894	0.026
14	*β*_*0*_ + *b*_j_ + Shrub height	4	322.183	3.937	0.026
15	*β*_*0*_ + *b*_j_ + Plant species richness	4	322.402	4.156	0.023
16	*β*_*0*_ + *b*_j_ + Woody biomass	4	322.670	4.424	0.020

a Both main effects and their interaction were fitted.

When both species were modeled together, including species as an additional categorical explanatory variable, 5 of the 24 candidate models (Appendix S4) were included in the 95% confidence set (Table 5). Model 1 had majority support to predict the differences in oryx and gazelle density (ΔAICc < 2) across the landscape, with the differences attributed to rock cover and plant species richness.

**Table 5:** The confidence set (cumulative *w*_i_ ≥ 0.95) and global null model for dorcas gazelle and scimitar‐horned oryx combined (based on 760 pellet events), including species as an additional categorical explanatory variable, with the number of parameters (*k*), AICc, ΔAICc, and Akaike weights (*w*_i_). The density model included the covariates, in addition to the intercept *β*_*0*_ and the random effect *b*_*j*_ (wadi system)

ID	Density model	*k*	AICc	ΔAICc	*w*_i_
1	*β*_*0*_ + *b*_j_ + Rock cover + Plant species richness	5	670.333	0.000	0.571
2	*β*_*0*_ + *b*_j_ + Rock cover + Habitat type (wadi/plain)	5	672.842	2.509	0.163
3	*β*_*0*_ + *b*_j_ + Rock cover + Ungulate species	5	672.844	2.511	0.163
4	*β*_*0*_ + *b*_j_ + Rock cover + Herbaceous height	5	675.356	5.023	0.046
5	*β*_*0*_ + *b*_j_ + Rock cover + Herbaceous cover	5	675.671	5.338	0.040
6	*β*_*0*_ + *b*_j_ (Global null model)	3	710.192	39.858	0.000

### A priori habitat classification

**Figure 2:**
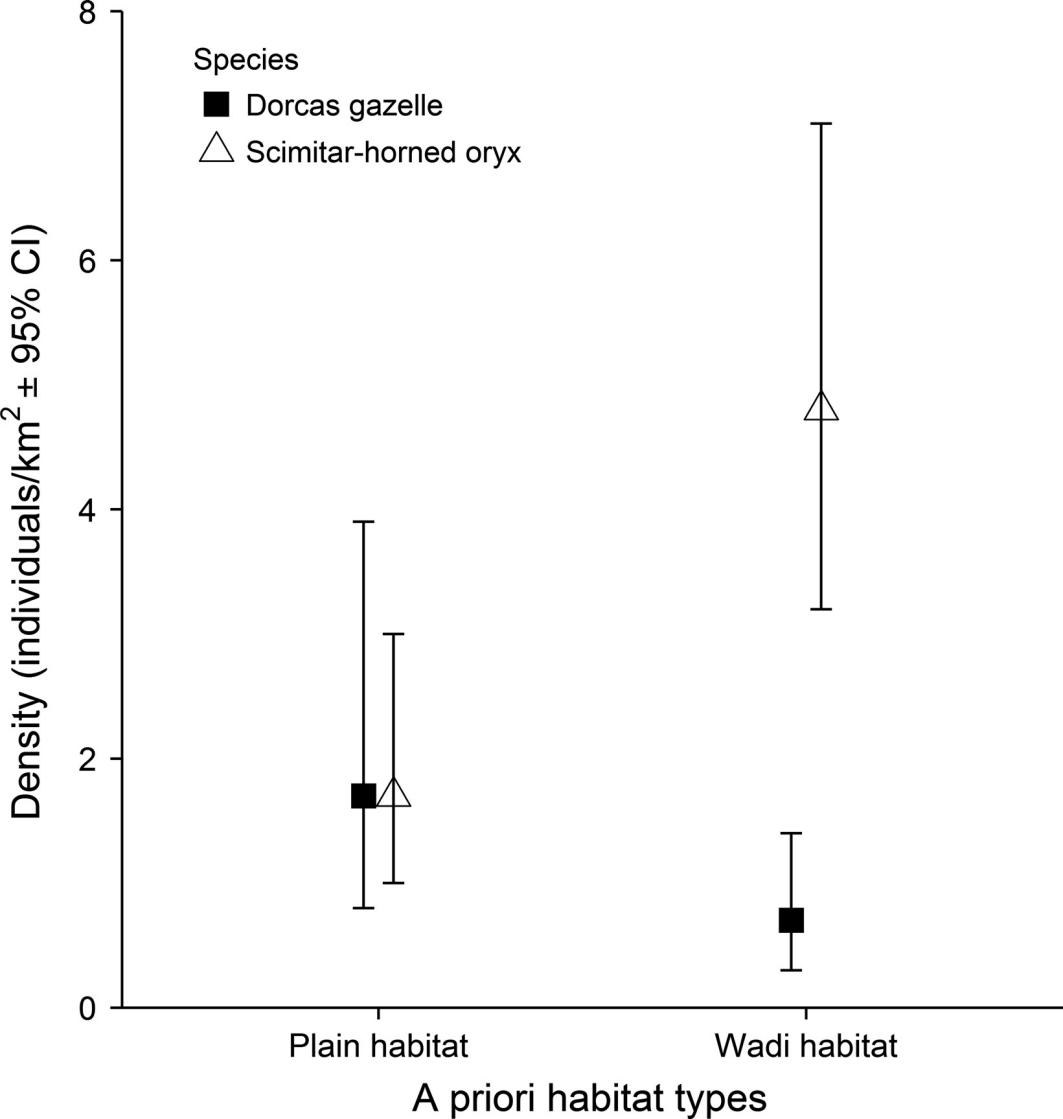
Indirect density estimates and 95% confidence intervals produced in DISTANCE, stratified by a priori habitat.

All habitat characteristics were higher in the wadi habitat (Wilcoxon rank‐sum test, *P* < 0.01), except for rock cover, which was lower (*W *=* *78.5, *P* < 0.01; Appendix S5). Oryx density was higher for the wadi habitat (Fig. 2; Appendix S6) with a selection ratio of 0.74, implying that oryx were three times more likely to select the wadis than the plain. The reverse was true for gazelle, with a selection ratio of 0.29 for the wadi and 0.71 for the plain.

### A posteriori habitat classification

**Figure 3:**
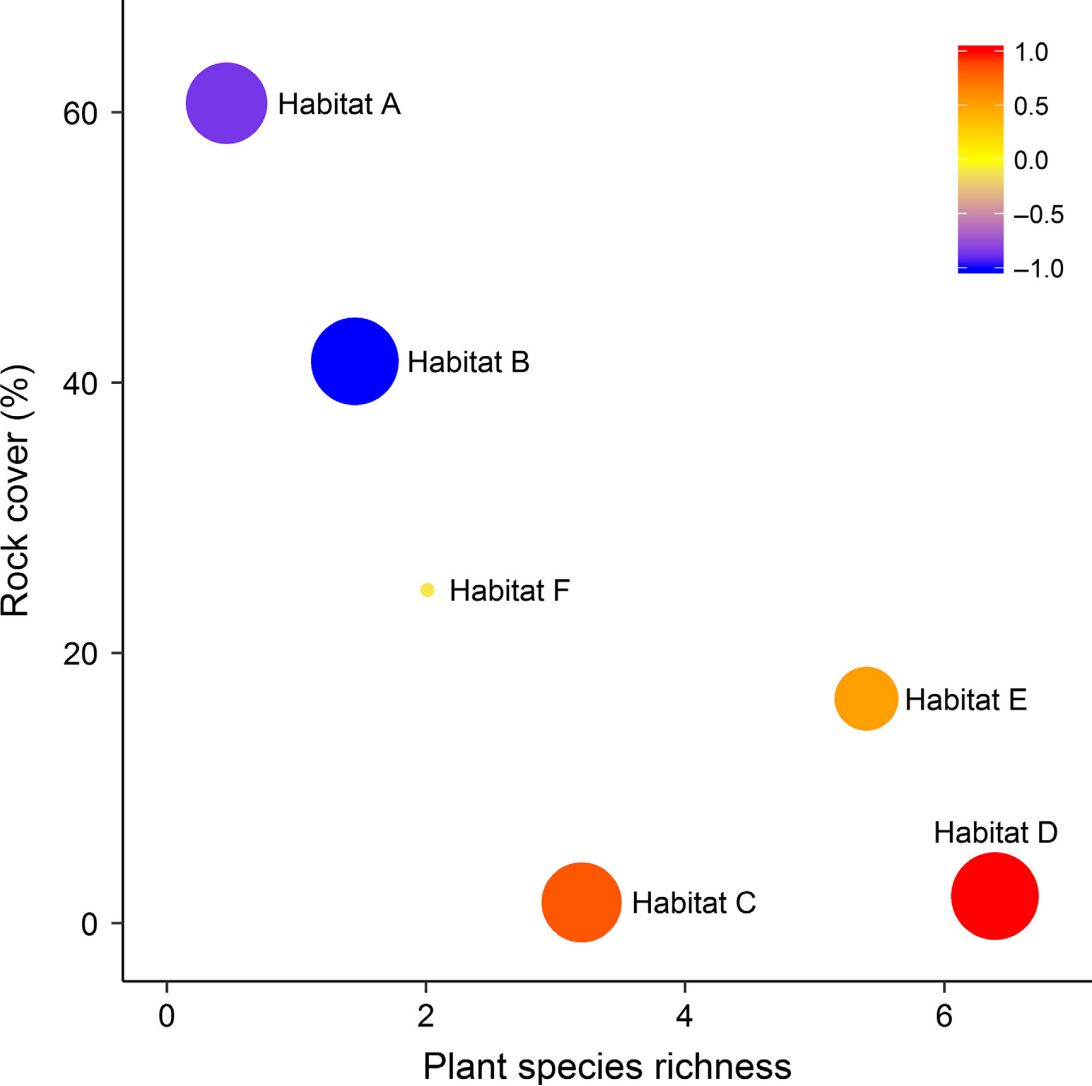
A posteriori habitat types with resource partitioning ratio (size) and combined density index (color) for scimitar‐horned oryx and dorcas gazelle. Point size is proportional to the ratio between oryx and gazelle density (small size indicates a shared habitat, and large size indicates a partitioned habitat), and color represents the density of oryx minus gazelle (red representing oryx dominance and blue gazelle dominance). A posteriori habitats are based on cluster analysis (Appendix S5), the difference in oryx and gazelle density, and the key predictor variables: rock cover and plant species richness.

The cluster analysis indicated that six habitats were distinguishable (Appendix S5) and showed separation on the axes of plant species richness and rock cover (Fig. 3). The habitats were defined as follows: (A) rocky plains with very sparse vegetation (plant cover <1.5%); (B) rocky plains with sparse vegetation (<3%); (C) sand dunes with intermediate vegetation (<20%); (D) densely vegetated (>50%) dune wadis, dominated by herbaceous cover of *Stipagrostis spp.;* and (E) densely vegetated (>40%) wadis characterized by *Retama raetam*. The final group (F) is a complex conglomerate of wadi and plain habitat with intermediate vegetation density (<20%). These habitats summarize the landscape from an ungulate's perspective and reveal the patterns of resource partitioning between gazelle and oryx in finer resolution (Fig. 3).

**Figure 4:**
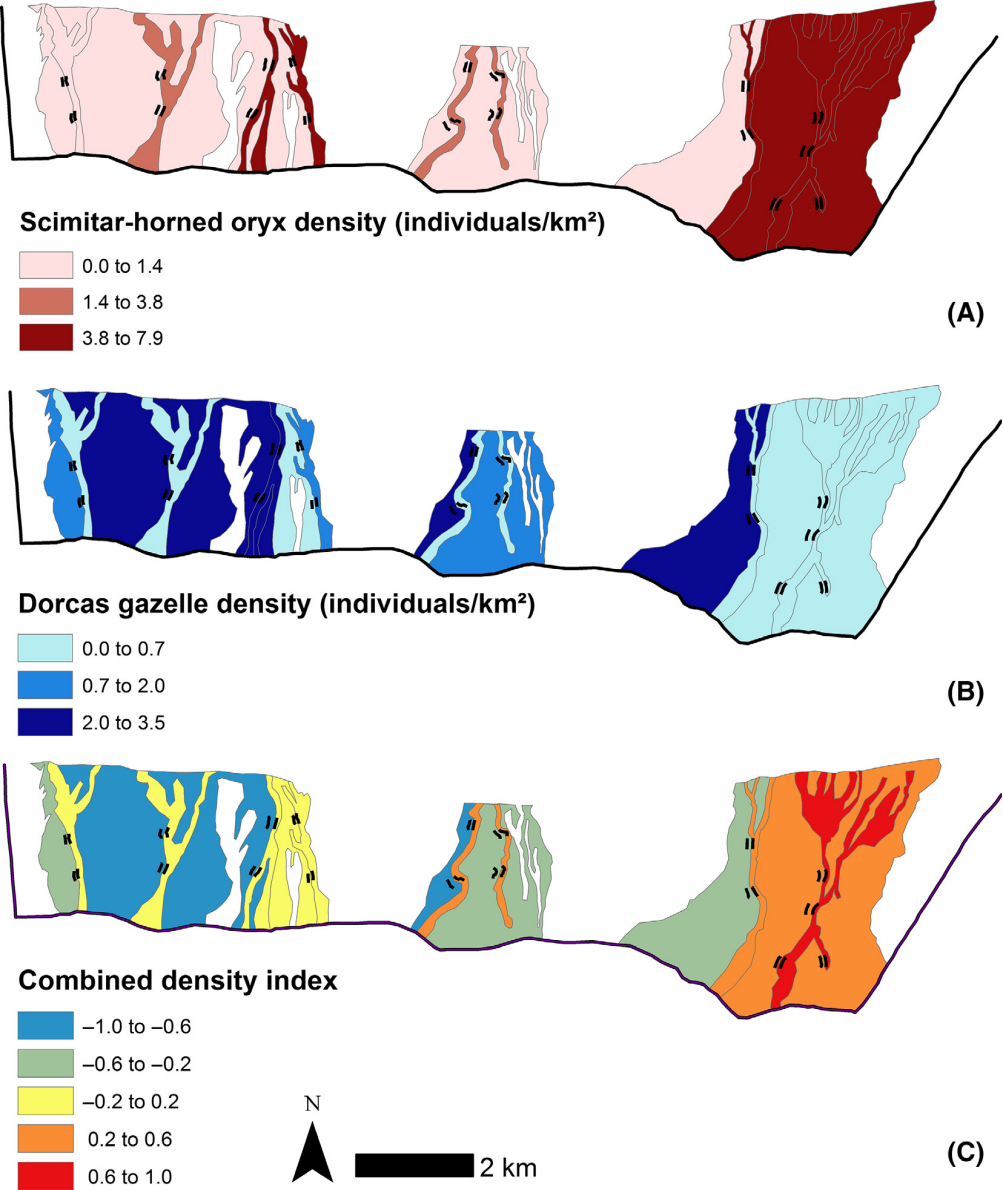
Spatial distribution of scimitar‐horned oryx (A), dorcas gazelle (B), and both species combined (C; density of oryx minus gazelle, red representing oryx dominance, blue gazelle dominance and yellow shared habitats) per habitat patch (as defined by the a priori approach). White represents unsampled regions (no transect located within the patch) where density is unknown.

Both species demonstrated spatial and resource selection, resulting in nonrandom, but overlapping distributions (Fig. 4). Overall, oryx selected strongest for habitat D (selection ratio 0.54: key resource area), followed by habitats C and E (0.24 and 0.13 respectively), whereas gazelle selected habitat B (0.61: key resource area), followed by A (0.14). The diversity of habitat use was effectively equal for oryx (H' = 0.55) and gazelle (H' = 0.51). These patterns can be surmised by three subresponses (Fig. 3): oryx dominated dune and wadi habitats (habitats C, D and E); gazelle dominated rocky plains (A and B); and shared resource use of the intermediate habitat (F). Although habitats may appear homogenous such as rocky plains, they may function very differently for the focal species. Gazelle were four times as likely to select rocky plains with sparse vegetation (habitat B) than very sparse vegetation (habitat A).

## Discussion

This is the first time that resource partitioning between ungulates has been studied using an integrated likelihood approach (Oedekoven et al. 2013) for distance‐sampled density estimates. This allows the impacts of explanatory variables to be tested in a regression framework. The approach provides a time‐averaged (over a period of 490–520 days) overview of the typical state of the gazelle‐oryx interrelationship in Dghoumes National Park, which is crucial for improving knowledge of the focal species' ecology and thus for enhancing conservation actions in poorly understood arid systems (Scillitani et al. 2013). We show that oryx and gazelle partition resources on the habitat axis, despite nondiscrete spatial distributions: oryx select for the wadi habitats and gazelle select for the open plain habitats (Fig. 2). This resource partitioning and high environmental heterogeneity facilitate coexistence between these species (Godsoe et al. 2015) and suggest differences in their realized niches through ecological competition. Temporal shifts in habitat selection could not be distinguished from this single time horizon. We may therefore underestimate the role of opportunistic resource use. In addition, the proximity of the plain to the wadi habitat transects could have influenced the results, although the strong partitioning identified suggests the habitats were selected independently by the focal species (Fig. 2). This could be further investigated in the future by placing plain transects at multiple distances away from the wadi transects. Our results nonetheless support the finding that gazelle select open areas and that habitat structure is the most important factor in selection (Abáigar et al. 2013). In particular, we extend those findings to show how vegetation density, as indicated by herbaceous height, litter cover, and herbaceous cover (Table 1), influenced use of resources and habitat by gazelle (Table 4).

Food quality and quantity have been suggested as the two main niche axes that allow resource partitioning for savannah ungulates (Cromsigt and Olff 2006). This was indirectly identified in arid environments, with oryx (Table 3) and Asiatic wild ass (Henley et al. 2007) selecting high plant species richness, which acts as a proxy for food quality (Table 1). This association may be part of a positive feedback loop, where large herbivores maximize nutrient intake by selecting a wide range of forage species (Freeland and Janzen 1974; Westoby 1978). These larger, less selectively feeding herbivores are known to increase plant diversity, due to their impact on dominant species and disturbance of the vegetation canopy (Bakker and Olff 2003). This relationship supports the prediction that the physiological need to consume a high diversity of plants may be particularly acute in deserts due to the low nutritional quality of arid plants (Noy‐Meir 1973; Herms and Mattson 1994). This ecophysiological need scales allometrically, being more important for the larger‐bodied oryx than the small‐bodied gazelle, leading to the wide dietary breadths of arid‐adapted ungulates (Owen‐Smith 1985).

Unlike Abáigar et al. (2013), we found no evidence that direct predation pressure from golden jackal is a driver of habitat selection in gazelle (*w*_i_ = 0.012; Appendix S4). However, predation risk/fear (Brown et al. 1999) might lead indirectly to the converse relationships with vegetation density (herbaceous cover and height) shown by gazelle and oryx. Sinclair et al. (2003) suggested a threshold body mass equal to that of the oryx marking a transition from predator‐limited to resource‐limited population dynamics, with gazelle experiencing higher predation pressure due to its smaller size and therefore selecting for more open habitat. Most large predators were extirpated from Tunisia prior to 1960, including cheetah (*Acinonyx jubatus*), which was the major predator of gazelle. Although this is over 11 gazelle generations ago (generation length = 4.9 years; IUCN in press), their preference for open habitats may nonetheless be driven by the “ghost of predation past,” in which antipredator behavior is maintained even after selection for it has relaxed (Byers 1997).

We therefore suggest that the wadi/plain partitioning could be driven by a combination of predator‐driven gazelle dynamics and resource‐limited oryx habitat selection. Targeted data collection would help elucidate the interplay between biotic and abiotic drivers of ungulate resource partitioning in arid environments.

The spatial distribution and resource selection ratios for oryx and gazelle illustrate their reliance on key resource areas (Figs. 3, 4). Oryx favored dune wadis dominated by herbaceous cover; gazelle selected rocky plains with sparse vegetation (Fig. 3). Gazelle showed weaker selection for habitat types, reflecting its more generalist strategy (Kingdon et al. 2013). These key resource areas, in combination with spatial heterogeneity, have the potential to buffer against temporal variability, and therefore major stochastic threats, including frequent episodic mortalities (Illius and O'Connor 2000; Cromsigt et al. 2009). The diversity of habitat use was lower for these arid‐adapted species (mean H' = 0.53) than for savannah species (mean H' ≈ 1; Cromsigt et al. 2009). This specific resource dependence indicates greater vulnerability to threats such as environmental change and/or homogenizing process (e.g., overstocking; Cromsigt et al. 2009). Conservation management should therefore prioritize resource‐limited species (Martin 2014), particularly the oryx. Limited protected area size exacerbates these threats and poses additional challenges, including disruption of migration/dispersal pathways, restricted access to seasonal forage, and the degradation of key resource areas (Durant et al. 2015). Translocations, for example, the proposed Tunisian meta‐population strategy (Woodfine et al. 2009), could help mitigate against this greater vulnerability to environmental change of fenced rather than free‐living populations, especially during times of drought or stress (Islam et al. 2010; Durant et al. 2015).

Reintroductions of ungulates are an important conservation component in North Africa (Abáigar et al. 2013), as the region has suffered a catastrophic decline in megafauna (Durant et al. 2014). Scillitani et al. (2013) state that identifying the factors driving resource selection by reintroduced species is crucial for improving conservation programs and this is a key application of our research. We have highlighted the role of habitat structure and nutrient availability and have demonstrated that although habitats may appear homogenous such as rocky plains, they may function differently for the focal species. This differential use reinforces the consensus that the persistence of a reintroduced population depends upon a complex suite of factors, and not just on food availability (Armstrong and Seddon 2008). Following reintroduction, both oryx and gazelle have demonstrated evidence of resource selection, distributing themselves according to their biological and behavioral preferences. This illustrates their ability to recover natural behaviors in a constrained environment and indicates their preferred resources postrelease (Abáigar et al. 2013). The selection of high‐quality resources is fundamental to individuals because it facilitates superior body condition and therefore the probability of reproduction and survival (Gaillard et al. 2010), a key step in any successful reintroduction program (Scillitani et al. 2013). We reveal the role of food quality for large ungulates (Tables 3, 5) and consider adequate plant species richness a prerequisite for future reintroductions to arid environments, for example, the proposal for a wild population of oryx in the Ouadi Rimé‐Ouadi Achim Reserve in Chad (Bemadjim et al. 2012). We also provide evidence that substrate (sand to rock) and habitat (open to closed) diversity are required to maintain a multiungulate system in an arid environment by facilitating niche separation (Godsoe et al. 2015).

Studying the ecology of arid species is important for conservation as they can reveal alternative relationships to savannah species and experience climatic extremes that generate sharp ecological gradients (Schulz et al. 2009). Our evidence from Dghoumes supports the prediction that habitat selection decisions are taken at various scales (plant, habitat, landscape) in arid environments (Henley et al. 2007). Oryx and gazelle showed differing selection of environmental covariates (Tables 3, 4), which lead to opposing selection at the habitat scale (Fig. 2). The strong selection for and against rock cover and against and for plant species richness, for gazelle and oryx, respectively, generates an axis of habitat selection differentiation between the species across the landscape (Figs. 3, 4; Table 5). Such habitat selection can provide important insights into species vulnerability in a rapidly changing environment and therefore their current and future extinction risk (Cromsigt et al. 2009; Martin 2014). Our results highlight the importance of adequate plant species richness for the overall ecosystem (Fig. 3; Table 5). The ability to identify similar limiting resources within other sites is essential for sustaining and reintroducing viable populations of threatened species in the expanding arid environments of the future.

## Conflict of Interest

None declared.

## Data Accessibility

Data available from figshare: https://figshare.com/articles/Resource_partitioning_between_fenced_ungulate_populations_in_arid_environments/1508558.

## Supporting information


**Appendix S1.** R code for a generalised linear mixed‐effect model via a log‐link with a Poisson error structure following the integrated likelihood approach.

**Appendix S2.** Estimating defecation rates for the focal species.
**Appendix S3.** Estimated and prospective decay rates for the focal species.
**Appendix S4.** Candidate models for scimitar‐horned oryx, dorcas gazelle and both species combined.
**Appendix S5.** A posteriori habitat types based on cluster‐analysis and habitat characteristics.
**Appendix S6.** Density estimates and 95% confidence intervals produced in DISTANCE for the a priori and a posteriori habitats.
