# Supplementary material for: Resource partitioning between ungulate populations in arid environments
Source: Ecol Evol. 2016 Aug 17;6(17):6354–65. doi: 10.1002/ece3.2218 (PMC5016655; doi:10.1002/ece3.2218)
Supplement: Supplementary file 1 — Appendix S1. R code for a generalised linear mixed‐effect model via a log‐link with a Poisson error structure following the integrated likelihood approach. [file ECE3-6-6354-s001.docx]

Appendix S1. R code for a generalised linear mixed-effect model via a log-link with a Poisson error structure following the integrated likelihood approach

####################################################################

# Robert Cooke #

# accompanies Cooke et al. 2016 (Resource partitioning between ungulate populations in arid environments) Ecology and Evolution #

# Adaptation of the integrated likelihood approach (Oedekoven et al. 2013) #

####################################################################

####################################################

# A. Preparation

####################################################

setwd("files_location")

# Set the working directory that is used for raiding/saving data tables

rm(list = ls())

# clear memory

or <- read.csv("file_name.csv")

# read in datasheet

head(or)

# dung data

# this is a data frame where each observed detection (in this case pellet event) is listed in a separate row along with associated logistic information and covariate values

# if no detection was made at a transect, one row is given with the logistic information and covariate values and NA’s for all columns dealing with the observation

qqnorm(or$Distance)

# Normality plot

####################################################

# B. Transform data and bin into 3 intervals

####################################################

or$cutdist <- cut(or$Distance, 3)

# bin data into 3 intervals

with(or, tapply(Distance, cutdist, length))

# number of pellet events in each interval

or$SitePair <- paste(or$Site, or$Pair, sep="-")

# Combine pairs and sites

j<-length(unique(or$SitePair))

Tj<-array(1,j)

# Tj is a vector of length(j)

Pair <- rep(c(1, 2), 36)

Site <- rep(rep(unique(or$Site), each=2),2)

# Set up Pair (habitat type) and site

Y <- with(or, tapply(SitePair, list(cutdist, SitePair), length))

Y[is.na(Y)] <- 0

Y1 <- t(Y[1,])

Y2 <- t(Y[2,])

Y3 <- t(Y[3,])

Y <- t(colSums(Y))

# matrices that will hold the counts for each site (1 row per site) for each of the three distance bands

(sum(Y1) + sum(Y2) + sum(Y3)) == length(or$Distance[!is.na(or$Distance)])

# Check that the sums of the three bins equal the length of the complete dataset

# should be TRUE

sum(Y[which(is.na(Y)==F)])

sum(Y1[which(is.na(Y)==F)]) + sum(Y2[which(is.na(Y)==F)]) + sum(Y3[which(is.na(Y)==F)])

# Another check to ensure bins and NAs are set up correctly

# Should be equal

####################################################

# C. Detection function

####################################################

xxx <- hist(or$Distance, main = "Detection frequency from centre-line", xlab = "Distance from centre-line (m)")

xxx

# understand detection frequency away from the centre-line

####################################################

# D. Likelihood functions for the detection model

####################################################

# hazard-rate function (pdf)

f.haz.function<-function(dis,sigma,shape) {

f <- 2*dis*(1-exp(-(dis/sigma)^(-shape)))

return(f)}

f.haz.int<-function(cutpoint1,cutpoint2,sigma,shape){

int.prob<-integrate(f.haz.function,cutpoint1,cutpoint2,sigma,shape)$value

return(int.prob)}

# half-nornmal function

f.hn.function<-function(dis,sigma){

f <- 2*dis*exp(-dis^2/(2*sigma^2))

return(f)}

f.hn.int<-function(cutpoint1,cutpoint2,sigma){

int.prob<-integrate(f.hn.function,cutpoint1,cutpoint2,sigma)$value

return(int.prob)}

# the area of the plot: length (220m) x width (8)

a <- 1760 # plot area

# cutpoints of the intervals (0, 1.33, 2.66, 4)

cutpoint <- seq(0, 12, 4)/3

####################################################

# E. Analysis

####################################################

########################## Model 1: Half-normal global

model1.log.lik.fct<-function(p){

sig1<-p[1]

int<-p[2]

std.ran<-exp(p[3])

# model parameters

fe<-array(NA,3)

for (im in 1:3){

fe[im]<-f.hn.int(cutpoint[im],cutpoint[im+1],sig1)/a}

# half-normal likelihood function

lik<-array(NA,j)

for (ik in 1:j){

lik[ik]<-integrate(function(bj){

# the product of all the observations

Tj.m<-Tj[ik]

l<-length(bj)

obs.prob1<-matrix(NA,Tj.m,l)

obs.prob2<-matrix(NA,Tj.m,l)

obs.prob3<-matrix(NA,Tj.m,l)

# 3 for the three distance bands

for (tk in 1:Tj.m){

lambda1<-exp(int + bj) * fe[1]

obs.prob1[tk,]<-dpois(Y1[ik],lambda1)

lambda2<-exp(int + bj) * fe[2]

obs.prob2[tk,]<-dpois(Y2[ik],lambda2)

lambda3<-exp(int + bj) * fe[3]

obs.prob3[tk,]<-dpois(Y3[ik],lambda3)}

obs.sum<-apply(obs.prob1,2,prod)*apply(obs.prob2,2,prod)*apply(obs.prob3,2,prod)*dnorm(bj,0,std.ran)

return(obs.sum)},-Inf,Inf)$value}

post<-sum(log(lik))

return(-post)}

model1.log.lik.fct(c(1.8, 8.0, log(0.5)))

# Maximum likelihood estimates (MLE) for the detection function, intercept (ß0) and the random effect (bj)

model1<-optim(par=c(1.8, 8.0, log(0.5)),model1.log.lik.fct,hessian=TRUE)

# optimising the likelihood function and calculating the hessian matrix (fitting the model)

model1.se<-sqrt(diag(solve(model1$hessian)))

model1.se

# analytical standard errors (ASEs) retrieved from the hessian matrix

model1

# model summary

np1 <- length(model1$par)

2*model1.log.lik.fct(model1$par) + 2*np1

# AIC

2*model1.log.lik.fct(model1$par) + 2*np1 + (2*np1*(np1+1))/(dim(or)[1] - np1 - 1)

# corrected AIC (AICc)

########################## Model 2: Hazard-rate global

model2.log.lik.fct<-function(p){

sig1<-p[1]

int<-p[2]

std.ran<-exp(p[3])

sha2<-2

# hazard detection shape

fe<-array(NA,3)

for (im in 1:3){

fe[im]<-f.haz.int(cutpoint[im],cutpoint[im+1],sig1,sha2)/a}

# hazard-rate likelihood function

lik<-array(NA,j)

for (ik in 1:j){

lik[ik]<-integrate(function(bj){

# the product of all the observations

Tj.m<-Tj[ik]

l<-length(bj)

obs.prob1<-matrix(NA,Tj.m,l)

obs.prob2<-matrix(NA,Tj.m,l)

obs.prob3<-matrix(NA,Tj.m,l)

# 3 for the three distance bands

for (tk in 1:Tj.m){

lambda1<-exp(int + bj) * fe[1]

obs.prob1[tk,]<-dpois(Y1[ik],lambda1)

lambda2<-exp(int + bj) * fe[2]

obs.prob2[tk,]<-dpois(Y2[ik],lambda2)

lambda3<-exp(int + bj) * fe[3]

obs.prob3[tk,]<-dpois(Y3[ik],lambda3)}

obs.sum<-apply(obs.prob1,2,prod)*apply(obs.prob2,2,prod)*apply(obs.prob3,2,prod)*dnorm(bj,0,std.ran)

return(obs.sum)},-Inf,Inf)$value}

post<-sum(log(lik))

return(-post)}

model2.log.lik.fct(c(12, 7, log(0.5)))

model2<-optim(par=c(12, 7, log(0.5)),model2.log.lik.fct,hessian=TRUE)

model2.se<-sqrt(diag(solve(model2$hessian)))

model2.se

# ASEs

model2

# model summary

np2 <- length(model2$par)

2*model2.log.lik.fct(model2$par) + 2*np2

# AIC

2*model2.log.lik.fct(model2$par) + 2*np2 + (2*np2*(np2+1))/(dim(or)[1] - np2 - 1)

# AICc

############### Model 3: Half-normal global with Pair (habitat type)

model3.log.lik.fct<-function(p){

sig1<-p[1]

int<-p[2]

pa<-p[3]

std.ran<-exp(p[4])

fe<-array(NA,3)

for (im in 1:3){

fe[im]<-f.hn.int(cutpoint[im],cutpoint[im+1],sig1)/a}

lik<-array(NA,j)

for (ik in 1:j){

lik[ik]<-integrate(function(bj){

# the product of all the observations

Tj.m<-Tj[ik]

l<-length(bj)

obs.prob1<-matrix(NA,Tj.m,l)

obs.prob2<-matrix(NA,Tj.m,l)

obs.prob3<-matrix(NA,Tj.m,l)

# 3 for the three distance bands

for (tk in 1:Tj.m){

lambda1<-exp(int + bj + Pair[ik]*pa) * fe[1]

obs.prob1[tk,]<-dpois(Y1[ik],lambda1)

lambda2<-exp(int + bj + Pair[ik]*pa) * fe[2]

obs.prob2[tk,]<-dpois(Y2[ik],lambda2)

lambda3<-exp(int + bj + Pair[ik]*pa) * fe[3]

obs.prob3[tk,]<-dpois(Y3[ik],lambda3)}

obs.sum<-apply(obs.prob1,2,prod)*apply(obs.prob2,2,prod)*apply(obs.prob3,2,prod)*dnorm(bj,0,std.ran)

return(obs.sum)},-Inf,Inf)$value}

post<-sum(log(lik))

return(-post)}

model3.log.lik.fct(c(1.8, 10.2, -1.5, log(0.5)))

# MLE for the detection function, intercept (ß0), the covariate Pair (Habitat type) and the random effect (bj)

model3<-optim(par=c(1.8, 10.2, -1.5, log(0.5)),model3.log.lik.fct,hessian=TRUE)

model3.se<-sqrt(diag(solve(model3$hessian)))

model3.se

# ASEs

model3

# model summary

np3 <- length(model3$par)

2*model3.log.lik.fct(model3$par) + 2*np3

# AIC

2*model3.log.lik.fct(model3$par) + 2*np3 + (2*np3*(np3+1))/(dim(or)[1] - np3 - 1)

# AICc

########### Model 13: Half-normal global with Plant species richness

or$sitspec <- paste(or$Site, or$Pair, or$Species.richness, sep="-")

Sprich <- as.numeric(with(or, tapply(Species.richness, sitspec, mean)))

# set up plant species richness covariate to match distance data

model13.log.lik.fct<-function(p){

sig1<-p[1]

int<-p[2]

spr<-p[3]

std.ran<-exp(p[4])

fe<-array(NA,3)

for (im in 1:3){

fe[im]<-f.hn.int(cutpoint[im],cutpoint[im+1],sig1)/a}

lik<-array(NA,j)

for (ik in 1:j){

lik[ik]<-integrate(function(bj){

# the product of all the observations

Tj.m<-Tj[ik]

l<-length(bj)

obs.prob1<-matrix(NA,Tj.m,l)

obs.prob2<-matrix(NA,Tj.m,l)

obs.prob3<-matrix(NA,Tj.m,l)

# 3 for the three distance bands

for (tk in 1:Tj.m){

lambda1<-exp(int + bj + Sprich[ik]*spr) * fe[1]

obs.prob1[tk,]<-dpois(Y1[ik],lambda1)

lambda2<-exp(int + bj + Sprich[ik]*spr) * fe[2]

obs.prob2[tk,]<-dpois(Y2[ik],lambda2)

lambda3<-exp(int + bj + Sprich[ik]*spr) * fe[3]

obs.prob3[tk,]<-dpois(Y3[ik],lambda3)}

obs.sum<-apply(obs.prob1,2,prod)*apply(obs.prob2,2,prod)*apply(obs.prob3,2,prod)*dnorm(bj,0,std.ran)

return(obs.sum)},-Inf,Inf)$value}

post<-sum(log(lik))

return(-post)}

model13.log.lik.fct(c(1.6, 7.2, 0.4, log(0.5)))

model13<-optim(par=c(1.6, 7.2, 0.4, log(0.5)),model13.log.lik.fct,hessian=TRUE)

model13.se<-sqrt(diag(solve(model13$hessian)))

model13.se

# ASEs

model13

# model summary

np13 <- length(model13$par)

2*model13.log.lik.fct(model13$par) + 2*np13

# AIC

2*model13.log.lik.fct(model13$par) + 2*np13 + (2*np13*(np13+1))/(dim(or)[1] - np13 - 1)

# AICc

################################# Model 19: Species richness + Rock

# Define rock

or$sitspec <- paste(or$Site, or$Pair, or$Rock, sep="-")

Rock <- as.numeric(with(or, tapply(Rock, sitspec, mean)))

model19.log.lik.fct<-function(p){

sig1<-p[1]

int<-p[2]

sp<-p[3]

rk<-p[4]

std.ran<-exp(p[5])

fe<-array(NA,3)

for (im in 1:3){

fe[im]<-f.hn.int(cutpoint[im],cutpoint[im+1],sig1)/a}

lik<-array(NA,j)

for (ik in 1:j){

lik[ik]<-integrate(function(bj){

# the product of all the observations

Tj.m<-Tj[ik]

l<-length(bj)

obs.prob1<-matrix(NA,Tj.m,l)

obs.prob2<-matrix(NA,Tj.m,l)

obs.prob3<-matrix(NA,Tj.m,l)

# 3 for the three distance bands

for (tk in 1:Tj.m){

lambda1<-exp(int + bj + Sprich[ik]*sp + Rock[ik]*rk) * fe[1]

obs.prob1[tk,]<-dpois(Y1[ik],lambda1)

lambda2<-exp(int + bj + Sprich[ik]*sp + Rock[ik]*rk) * fe[2]

obs.prob2[tk,]<-dpois(Y2[ik],lambda2)

lambda3<-exp(int + bj + Sprich[ik]*sp + Rock[ik]*rk) * fe[3]

obs.prob3[tk,]<-dpois(Y3[ik],lambda3)}

obs.sum<-apply(obs.prob1,2,prod)*apply(obs.prob2,2,prod)*apply(obs.prob3,2,prod)*dnorm(bj,0,std.ran)

return(obs.sum)},-Inf,Inf)$value}

post<-sum(log(lik))

return(-post)}

model19.log.lik.fct(c(1.8, 8.2, 0.2, -0.03, log(0.5)))

model19<-optim(par=c(1.8, 8.2, 0.2, -0.03, log(0.5)),model19.log.lik.fct,hessian=TRUE)

model19.se<-sqrt(diag(solve(model19$hessian)))

model19.se

# ASEs

model19

# model summary

np19 <- length(model19$par)

2*model19.log.lik.fct(model19$par) + 2*np19

# AIC

2*model19.log.lik.fct(model19$par) + 2*np19 + (2*np19*(np19+1))/(dim(or)[1] - np19 - 1)

# AICc
