# Supplementary material for: Resource partitioning between ungulate populations in arid environments
Source: Ecol Evol. 2016 Aug 17;6(17):6354–65. doi: 10.1002/ece3.2218 (PMC5016655; doi:10.1002/ece3.2218)
Supplement: Supplementary file 2 — Appendix S2. Estimating defecation rates for the focal species. Appendix S3. Estimated and prospective decay rates for the focal species. Appendix S4. Candidate models for scimitar‐horned oryx, dorcas gazelle and both species combined. Appendix S5. A posteriori habitat types based on cluster‐analysis and habitat characteristics. Appendix S6. Density estimates and 95% confidence intervals produced in DISTANCE for the a priori and a posteriori habitats. [file ECE3-6-6354-s002.docx]

## **Appendix S2. Estimating defecation rates for the focal species**

Daily defecation rates were estimated by placing a known number of animals in an enclosure, previously cleared of signs, and monitoring the number of signs produced over a fixed period (Laing et al. 2003). Ideally, defecation rates should be estimated for the population under consideration (Marques et al. 2001). However, this was not practical, so defecation rates were obtained from small captive groups of scimitar-horned oryx (Table S2a) and dorcas gazelle (Table S2b) housed at Marwell Zoo, UK and a group at Oued Dekouk National Park, Tunisia that were held within a temporary 100x100 m enclosure. Calves had a much lower defecation rate than the adult groups and are often excluded from estimates due to this reason, however from demographic records within Dghoumes National Park it was estimated that calves contribute 10% to the demography of the population.

Table S2a. Daily defecation rates for scimitar-horned oryx at Marwell Zoo (diet: pellets, hay and grass) and Oued Dekouk National Park, Tunisia (diet: hay and barley *ad libitum*)

|  | Number | | |  | Pellet events per individual per day |
| --- | --- | --- | --- | --- | --- |
| Session | Males | Females | Calves |  |  |
| June 2014, Marwell Zoo, UK | 5 | 0 | 0 | Median | 6 |
|  |  |  |  | Mean | 6.6 ±0.42 |
|  |  |  |  | Range | 6-9 |
| June 2014, Oued Dekouk, Tunisia | 2 | 14 | 0 | Median | 8.5 |
|  |  |  |  | Mean | 7.9 ±0.26 |
|  |  |  |  | Range | 5.5-9.8 |
| June 2014, Oued Dekouk, Tunisia | 0 | 0 | 10 | Median | 4.4 |
|  |  |  |  | Mean | 4.5 ±0.18 |
|  |  |  |  | Range | 3.3-5.7 |
| Combined^a^ | 7 | 14 | 2 | Median | 8.5 |
|  |  |  |  | Mean | 7.5 ±0.26 |
|  |  |  |  | Range | 4.5-9.8 |
| ^a^Combined estimates from Marwell and Oued Dekouk with calves added as a 10% proportion from the mean of a group of 10 calves | | | | | |

Table S2b. Daily defecation rates for dorcas gazelle at Marwell Zoo, UK (diet: hay and pellets)

|  | Number | | |  | Pellet events per individual per day |
| --- | --- | --- | --- | --- | --- |
| Session | Male | Female | Juvenile^a^ |  |  |
| June 2014, Marwell Zoo, UK | 2 | 3 | 1 | Median | 12.8 |
|  |  |  |  | Mean | 12.8 ±0.70 |
|  |  |  |  | Range | 9-14.8 |
| ^a^Individuals were considered juvenile if they were less than 1 year old (Yom-Tov et al. 1995) | | | | | |

## **Appendix S3. Estimated and prospective decay rates for the focal species**

A prospective approach was applied to the estimation of decay rate (Laing et al. 2003), whereby 52 and 40 pellet events for scimitar-horned oryx and dorcas gazelle respectively, were identified at time of initial survey. Return visits were then made approximately every 5 days until the pellet events completely decayed. Fresh pellet events were not targeted, as monitoring these generates an estimate of the life span of freshly deposited pellet events, rather than an estimate of the average life span of pellet events (Marques et al. 2001). Pellet events were considered decayed (<10 pellets remaining) when they had been covered by leaves, had been spread out over a large area as a result of trampling, or had undergone organic decay (Marques et al. 2001). During a 26-day period, the decay rate was found to be very low (less than 5%) (Fig. S3a), decay rate was therefore assumed negligible and was not included as a likelihood component in the models. However to obtain densities in DISTANCE a value for the decay rate in days is required. Unfortunately before at least 90% of pellet events had completely decayed an extreme rain event led to the majority of monitored pellet events being washed away. However, when the pellet events were first identified their age was estimated with assistance from the local guards. These age estimates were then modelled (Fig. S3b) to obtain approximate decay rates for scimitar-horned oryx (520 ±150 days) and dorcas gazelle (490 ±130 days). The validity of these decay rates was tested by carrying out sensitivity analysis within DISTANCE and it was found that density estimates were insensitive (<10% change) to variations in decay rate of up to 100 days. This approach does not include the number of pellet events that decayed before the survey started and therefore leads to upward bias. This bias leads to a cautious estimate of density, which is preferable.


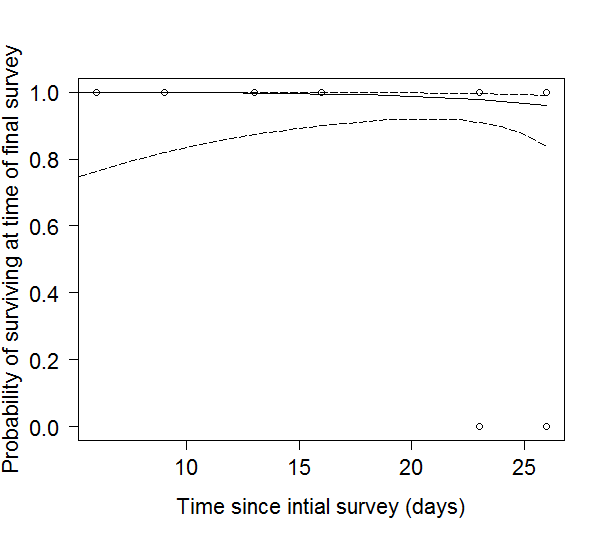

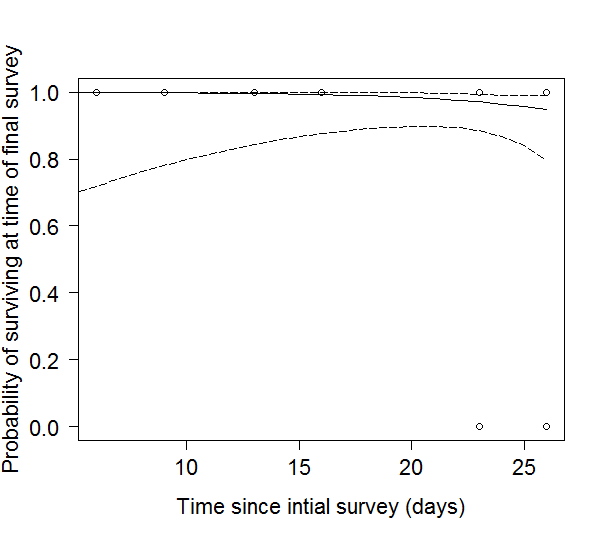


(a)

(b)

Figure S3a. Logistic regression curves of prospective decay rates for (a) scimitar-horned oryx (n = 52) and (b) dorcas gazelle (n = 40), with 95% confidence intervals (dashed lines). Points represent multiple pellet events.


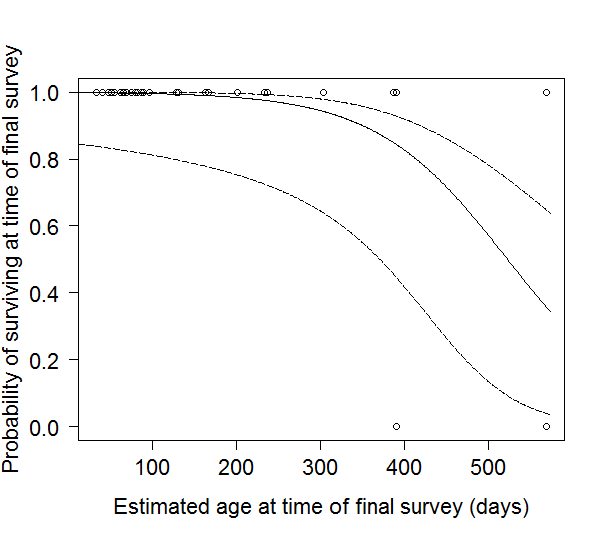

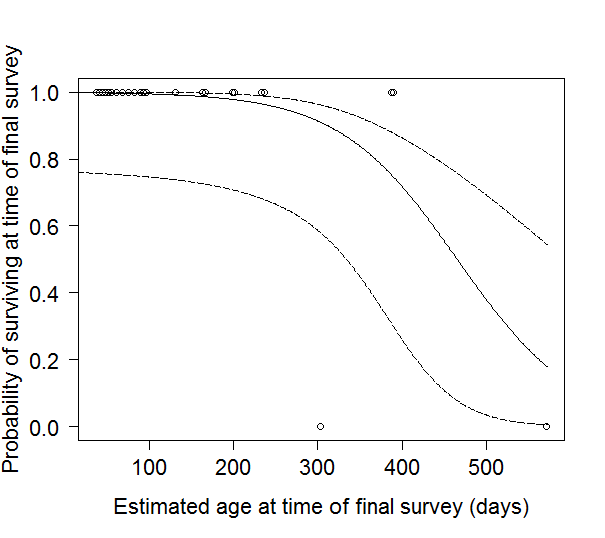


(a)

(b)

Figure S3b. Logistic regression curves of decay rates from estimated pellet event ages for (a) scimitar-horned oryx (n = 52) and (b) dorcas gazelle (n = 40), with 95% confidence intervals (dashed lines). Points represent multiple pellet events.

## **Appendix S4. Candidate models for scimitar-horned oryx, dorcas gazelle and both species combined**

Table S4a. The candidate set of models for scimitar-horned oryx (based on 628 pellet events), with the number of parameters (k), AICc, ΔAICc and Akaike weights (w_i_). The density model included the covariates, in addition to the intercept *β_0_* and the random effect *b_j_* (wadi system)

| ID | Density model | k | AICc | ΔAICc | w_i_ |
| --- | --- | --- | --- | --- | --- |
| 1 | *β_0_ + b_j_* + Rock cover + Plant species richness | 5 | 524.718 | 0.000 | 0.488 |
| 2 | *β_0_* + *b*_j_ + Rock cover + Habitat type (wadi/plain) | 5 | 525.626 | 0.901 | 0.310 |
| 3 | *β_0_* + *b*_j_ + Rock cover + Herbaceous height | 5 | 528.079 | 3.362 | 0.091 |
| 4 | *β_0_* + *b*_j_ + Rock cover + Herbaceous cover | 5 | 528.752 | 4.034 | 0.065 |
| 5 | *β_0_* + *b*_j_ + Rock cover | 4 | 529.644 | 4.927 | 0.042 |
| 6 | *β_0_* + *b*_j_ + Plant species richness | 4 | 533.766 | 9.048 | 0.005 |
| 7 | *β_0_* + *b*_j_ + Habitat type | 4 | 539.588 | 14.870 | 0.000 |
| 8 | *β_0_* + *b*_j_ + Herbaceous cover | 4 | 542.720 | 18.002 | 0.000 |
| 9 | *β_0_* + *b*_j_ + Herbaceous height | 4 | 543.395 | 18.678 | 0.000 |
| 10 | *β_0_* + *b*_j_ + Litter cover | 4 | 547.669 | 22.951 | 0.000 |
| 11 | *β_0_* + *b*_j_ + Tree height | 4 | 548.386 | 23.669 | 0.000 |
| 12 | *β_0_* + *b*_j_ + Shrub height | 4 | 548.546 | 23.828 | 0.000 |
| 13 | *β_0_* + *b*_j_ + Non-woody biomass | 4 | 550.880 | 26.162 | 0.000 |
| 14 | *β_0_* + *b*_j_ + Plant water content | 4 | 553.117 | 28.399 | 0.000 |
| 15 | *β_0_* + *b*_j_ + Tree cover | 4 | 554.583 | 29.865 | 0.000 |
| 16 | *β_0_* + *b*_j_ + Shrub cover | 4 | 554.760 | 30.042 | 0.000 |
| 17 | *β_0_* + *b*_j_ + North-south gradient | 4 | 555.688 | 30.970 | 0.000 |
| 18 | *β_0_* + *b*_j_ + East-west gradient | 4 | 555.854 | 31.136 | 0.000 |
| 19 | *β_0_* + *b*_j_ (Global null model) | 3 | 555.984 | 31.267 | 0.000 |
| 20 | *β_0_* + *b*_j_ + Predation | 4 | 556.594 | 31.877 | 0.000 |
| 21 | *β_0_* + *b*_j_ + Woody biomass | 4 | 557.367 | 32.649 | 0.000 |
| 22 | *β_0_* + *b*_j_ + Wadi location | 4 | 557.942 | 33.224 | 0.000 |

Table S4b. The candidate set of models for dorcas gazelle (based on 132 pellet events), with the number of parameters (k), AICc, ΔAICc and Akaike weights (w_i_). The density model included the covariates, in addition to the intercept *β_0_* and the random effect *b_j_* (wadi system)*.* * indicates that both main effects and their interaction were fitted

| ID | Density model | k | AICc | ΔAICc | w_i_ |
| --- | --- | --- | --- | --- | --- |
| 1 | *β_0_* + *b*_j_ + Herbaceous height * Litter cover | 6 | 318.246 | 0.000 | 0.184 |
| 2 | *β_0_* + *b*_j_ + Herbaceous height | 4 | 318.781 | 0.535 | 0.141 |
| 3 | *β_0_* + *b*_j_ + Litter cover | 4 | 319.157 | 0.911 | 0.117 |
| 4 | *β_0_* + *b*_j_ + Herbaceous cover | 4 | 319.265 | 1.019 | 0.111 |
| 5 | *β_0_* + *b*_j_ + Non-woody biomass | 4 | 320.477 | 2.231 | 0.060 |
| 6 | *β_0_* + *b*_j_ (Global null model) | 3 | 320.800 | 2.554 | 0.051 |
| 7 | *β_0_* + *b*_j_ + Plant water content | 4 | 320.933 | 2.687 | 0.048 |
| 8 | *β_0_* + *b*_j_ + East-west gradient | 4 | 321.591 | 3.345 | 0.035 |
| 9 | *β_0_* + *b*_j_ + Tree cover | 4 | 321.782 | 3.536 | 0.031 |
| 10 | *β_0_* + *b*_j_ + Habitat type (wadi/plain) | 4 | 321.890 | 3.644 | 0.030 |
| 11 | *β_0_* + *b*_j_ + North-south gradient | 4 | 321.905 | 3.659 | 0.030 |
| 12 | *β_0_* + *b*_j_ + Tree height | 4 | 322.089 | 3.843 | 0.027 |
| 13 | *β_0_* + *b*_j_ + Shrub cover | 4 | 322.140 | 3.894 | 0.026 |
| 14 | *β_0_* + *b*_j_ + Shrub height | 4 | 322.183 | 3.937 | 0.026 |
| 15 | *β_0_* + *b*_j_ + Plant species richness | 4 | 322.402 | 4.156 | 0.023 |
| 16 | *β_0_* + *b*_j_ + Woody biomass | 4 | 322.670 | 4.424 | 0.020 |
| 17 | *β_0_* + *b*_j_ + Wadi location | 4 | 323.175 | 4.929 | 0.016 |
| 18 | *β_0_* + *b*_j_ + Predation | 4 | 323.724 | 5.478 | 0.012 |
| 19 | *β_0_* + *b*_j_ + Rock cover | 4 | 323.894 | 5.648 | 0.011 |

Table S4c. The candidate set of models for dorcas gazelle and scimitar-horned oryx combined (based on 760 pellet events), including species as an additional categorical explanatory variable, with the number of parameters (k), AICc, ΔAICc and Akaike weights (w_i_). The density model included the covariates, in addition to the intercept *β_0_* and the random effect *b_j_* (wadi system)

| ID | Density model | k | AICc | ΔAICc | w_i_ |
| --- | --- | --- | --- | --- | --- |
| 1 | *β_0_* + *b*_j_ + Rock cover + Plant species richness | 5 | 670.333 | 0.000 | 0.571 |
| 2 | *β_0_* + *b*_j_ + Rock cover + Habitat type | 5 | 672.842 | 2.509 | 0.163 |
| 3 | *β_0_* + *b*_j_ + Rock cover + Ungulate species | 5 | 672.844 | 2.511 | 0.163 |
| 4 | *β_0_* + *b*_j_ + Rock cover + Herbaceous height | 5 | 675.356 | 5.023 | 0.046 |
| 5 | *β_0_* + *b*_j_ + Rock cover + Herbaceous cover | 5 | 675.671 | 5.338 | 0.040 |
| 6 | *β_0_* + *b*_j_ + Rock cover | 4 | 677.402 | 7.068 | 0.017 |
| 7 | *β_0_* + *b*_j_ + Plant species richness | 4 | 684.098 | 13.765 | 0.001 |
| 8 | *β_0_* + *b*_j_ + Habitat type (wadi/plain) | 4 | 695.633 | 25.299 | 0.000 |
| 9 | *β_0_* + *b*_j_ + Ungulate species | 4 | 695.635 | 25.301 | 0.000 |
| 10 | *β_0_* + *b*_j_ + Herbaceous cover | 4 | 696.411 | 26.077 | 0.000 |
| 11 | *β_0_* + *b*_j_ + Herbaceous height | 4 | 699.512 | 29.178 | 0.000 |
| 12 | *β_0_* + *b*_j_ + Shrub height | 4 | 700.363 | 30.029 | 0.000 |
| 13 | *β_0_* + *b*_j_ + Litter cover | 4 | 701.530 | 31.197 | 0.000 |
| 14 | *β_0_* + *b*_j_ + Tree height | 4 | 703.791 | 33.458 | 0.000 |
| 15 | *β_0_* + *b*_j_ + Non-woody biomass | 4 | 704.438 | 34.105 | 0.000 |
| 16 | *β_0_* + *b*_j_ + Plant water content | 4 | 706.516 | 36.182 | 0.000 |
| 17 | *β_0_* + *b*_j_ + East-west gradient | 4 | 706.972 | 36.638 | 0.000 |
| 18 | *β_0_* + *b*_j_ + Shrub cover | 4 | 707.446 | 37.113 | 0.000 |
| 19 | *β_0_* + *b*_j_ + Tree cover | 4 | 708.878 | 38.554 | 0.000 |
| 20 | *β_0_* + *b*_j_ (Global null model) | 3 | 710.192 | 39.858 | 0.000 |
| 21 | *β_0_* + *b*_j_ + Predation | 4 | 711.242 | 40.909 | 0.000 |
| 22 | *β_0_* + *b*_j_ + Woody biomass | 4 | 711.804 | 41.470 | 0.000 |
| 23 | *β_0_* + *b*_j_ + North-south gradient | 4 | 711.941 | 41.608 | 0.000 |
| 24 | *β_0_* + *b*_j_ + Wadi location | 4 | 712.058 | 41.725 | 0.000 |

## **Appendix S5. *A posteriori* habitat types based on cluster-analysis and habitat characteristics**

Ward hierarchical clustering of the sampled transects based on their similarity in scimitar-horned oryx and dorcas gazelle density and the key predictor variables from the combined models (Table S4c): rock cover and plant species richness (Fig. S5a).


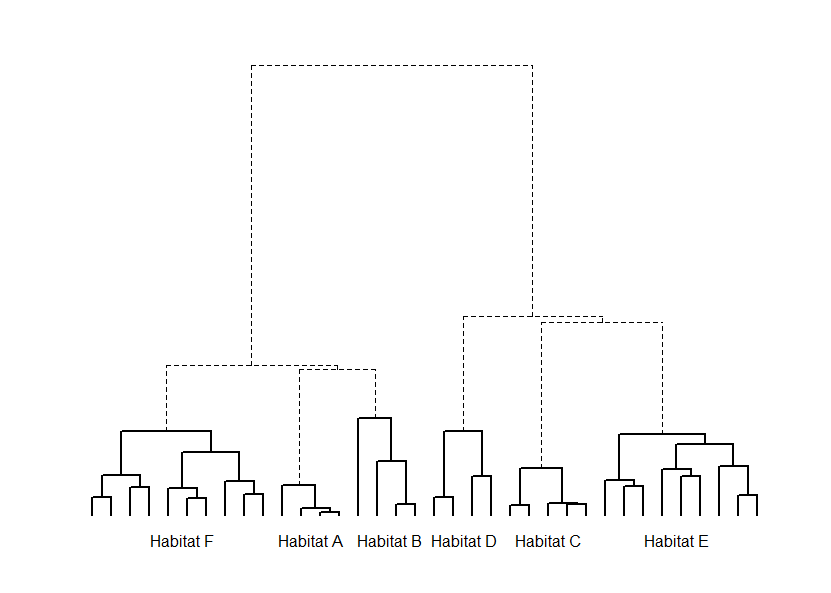


Figure S5a. Dendrogram showing *a posteriori* habitat types for each transect

To describe the differences between these habitat types numerically the mean values for the vegetative components for each *a priori* (Table S5a) and *a posteriori* habitats (Table S5b) are provided.

Table S5a. Mean and standard deviation for all quantified vegetation components for the *a priori* habitats. Number of transects per habitat type in brackets

|  | Habitat type | |
| --- | --- | --- |
|  | Wadi habitat (18) | Plain habitat (18) |
| Rock cover (%) | 12.12 ±2.69 | 33.51 ±5.57 |
| Litter cover (%) | 13.68 ±1.75 | 2.90 ±0.23 |
| Herbaceous cover (%) | 20.61 ±3.44 | 2.92 ±0.61 |
| Herbaceous height (cm) | 14.18 ±2.27 | 1.80 ±0.37 |
| Shrub cover (%) | 13.03 ±1.80 | 3.87 ±1.15 |
| Shrub height (cm) | 16.08 ±1.62 | 4.37 ±1.04 |
| Tree cover (%) | 7.01 ±1.86 | 0.10 ±0.10 |
| Tree height (cm) | 19.88 ±3.57 | 0.43 ±0.43 |
| Plant species richness | 5.00 ±0.39 | 1.54 ±0.24 |
| Woody biomass (g m^-2^) | 42.95 ±25.82 | 1.53 ±0.64 |
| Non-woody biomass (gDM m^-2^) | 22.70 ±6.88 | 1.59 ±0.56 |
| Water content (%) | 16.15 ±2.40 | 1.90 ±0.57 |

Table S5b. Mean and standard deviation for all quantified vegetation components for the *a posteriori* habitats. Number of transects per habitat type in brackets

|  | Habitat types | | | | | |
| --- | --- | --- | --- | --- | --- | --- |
|  | A (4) | B (4) | C (5) | D (4) | E (9) | F (10) |
| **Rock cover (%)** | **60.68 ±5.39** | **41.59 ±9.87** | **1.55 ±1.55** | **2.02 ±1.68** | **16.63 ±3.76** | **24.67 ±4.35** |
| Litter cover (%) | 2.27 ±0.16 | 2.61 ±0.37 | 4.27 ±0.25 | 20.77 ±3.39 | 12.07 ±2.12 | 6.59 ±2.10 |
| Herbaceous cover (%) | 0.43 ±0.25 | 1.86 ±0.40 | 7.40 ±1.06 | **40.00 ±8.35** | 18.33 ±2.69 | 5.25 ± 1.32 |
| Herbaceous height (cm) | 0.55 ±0.33 | 1.99 ±0.49 | 2.91 ±0.95 | 26.12 ±4.92 | 12.92 ±2.21 | 4.23 ±1.34 |
| Shrub cover (%) | 0.98 ±0.41 | 0.77 ±0.20 | 12.22 ±3.48 | 5.95 ±2.12 | 13.53 ±1.67 | 9.05 ±3.08 |
| Shrub height (cm) | 1.35 ± 0.53 | 2.19 ±0.83 | 10.88 ±2.45 | 11.56 ±2.22 | 18.37 ±2.31 | 8.80 ±2.56 |
| Tree cover (%) | 0.00 ±0.00 | 0.00 ±0.00 | 0.00 ±0.00 | 7.25 ±2.82 | 7.87 ±3.27 | 2.82 ±1.62 |
| Tree height (cm) | 0.00 ±0.00 | 0.00 ±0.00 | 0.00 ±0.00 | 23.93 ±3.27 | 21.21 ±6.25 | 7.90 ±3.61 |
| **Plant species richness** | **0.46 ±0.17** | **1.45 ±0.46** | **3.20 ±0.31** | **6.39 ±0.49** | **5.40 ±0.48** | **2.01 ±0.30** |
| Woody biomass (g m^-2^) | 0.09 ±0.09 | 0.00 ±0.00 | 4.66 ±2.39 | 7.93 ±3.38 | **39.95 ±32.37** | 8.55 ±4.19 |
| Non-woody biomass (gDM m^-2^) | 0.07 ±0.07 | 0.16 ±0.14 | 3.15 ±1.02 | **36.88 ±10.39** | 9.70 ±4.69 | 2.18 ±0.70 |
| Water content (%) | 0.30 ±0.30 | 0.87 ±0.87 | 4.10 ±1.13 | 12.14 ±4.23 | 17.63 ±3.88 | 9.25 ±3.26 |

## **Appendix S6. Density estimates and 95% confidence intervals produced in DISTANCE for the *a priori* and *a posteriori* habitats.**

Table S6a. Indirect density estimates and 95% confidence intervals in parentheses produced in DISTANCE, stratified by *a priori* habitat (based on a total of 760 pellet events)

|  | Habitat type | |
| --- | --- | --- |
|  | Wadi habitat | Plain habitat |
| Scimitar-horned oryx | 4.8 (3.2-7.1) | 1.7 (1.0-3.0) |
| Dorcas gazelle | 0.7 (0.3-1.4) | 1.7 (0.8-3.9) |

Table S6b. Indirect density estimates and 95% confidence intervals produced in DISTANCE, stratified by *a posteriori* habitat (based on a total of 760 pellet events)

|  | Habitat types | | | | | |
| --- | --- | --- | --- | --- | --- | --- |
|  | A | B | C | D | E | F |
| Scimitar-horned oryx | 0.3  (0.0-0.7) | 0.5  (0.1-1.0) | 4.7  (2.4-8.0) | 8.4  (4.8-13.0) | 4.6  (3.2-6.1) | 1.5  (1.0-2.0) |
| Dorcas gazelle | 0.8  (0.0-2.0) | 3.5  (1.7-5.9) | 0.4  (0.1-1.0) | 0.0  (0.0-0.0) | 1.3  (0.6-1.9) | 1.2  (0.5-2.5) |
